# Formation and Growth of Oligomers: A Monte Carlo Study of an Amyloid Tau Fragment

**DOI:** 10.1371/journal.pcbi.1000238

**Published:** 2008-12-05

**Authors:** Da-Wei Li, Sandipan Mohanty, Anders Irbäck, Shuanghong Huo

**Affiliations:** 1 Gustaf H. Carlson School of Chemistry and Biochemistry, Clark University, Worcester, Massachusetts, United States of America; 2 John von Neumann Institut für Computing, Forschungszentrum Jülich, Jülich, Germany; 3 Computational Biology and Biological Physics Division, Department of Theoretical Physics, Lund University, Lund, Sweden; National Cancer Institute, United States of America and Tel Aviv University, Israel

## Abstract

Small oligomers formed early in the process of amyloid fibril formation may be the major toxic species in Alzheimer's disease. We investigate the early stages of amyloid aggregation for the tau fragment AcPHF6 (Ac-VQIVYK-NH2) using an implicit solvent all-atom model and extensive Monte Carlo simulations of 12, 24, and 36 chains. A variety of small metastable aggregates form and dissolve until an aggregate of a critical size and conformation arises. However, the stable oligomers, which are β-sheet-rich and feature many hydrophobic contacts, are not always growth-ready. The simulations indicate instead that these supercritical oligomers spend a lengthy period in equilibrium in which considerable reorganization takes place accompanied by exchange of chains with the solution. Growth competence of the stable oligomers correlates with the alignment of the strands in the β-sheets. The larger aggregates seen in our simulations are all composed of two twisted β-sheets, packed against each other with hydrophobic side chains at the sheet–sheet interface. These β-sandwiches show similarities with the proposed steric zipper structure for PHF6 fibrils but have a mixed parallel/antiparallel β-strand organization as opposed to the parallel organization found in experiments on fibrils. Interestingly, we find that the fraction of parallel β-sheet structure increases with aggregate size. We speculate that the reorganization of the β-sheets into parallel ones is an important rate-limiting step in the formation of PHF6 fibrils.

## Author Summary

It is believed that the self association of certain protein molecules into aggregated structures, known as amyloid fibrils, plays an important role in a variety of human diseases, such as Alzheimer's disease and Parkinson's disease. Although the ability to form such amyloid fibrils is a common property for proteins, the process leading to these fibrils is incompletely understood. The early stages of the process involve small transient heterogeneous structures made of a few protein chains and are especially difficult to characterize. Here we use atomic-level simulations to explore the early part of the aggregation process for a fibril-forming fragment of the protein tau associated with Alzheimer's disease. We find that a multitude of small aggregates, rich in sheetlike structures, form through a nucleation process. Interestingly, a statistically preferred type of aggregate, consisting of two tightly packed sheets, emerges with increasing aggregate size. Growth of these larger aggregates seems to be a slow process that correlates with the emergence of more uniformly ordered sheets. We speculate that reorganization of the protein chains leading to that ordered arrangement is an important bottleneck to amyloid fibril formation for this peptide.

## Introduction

A century ago, Alois Alzheimer reported dense extracellular deposits and intracellular neuronal aggregates in the brain of a patient suffering from memory loss, focal symptoms, delusions, and hallucinations [1]. The extracellular deposits have been subsequently identified as amyloid plaques composed of an accumulation of β-amyloid peptides, while the intracellular neuronal aggregates are neurofibrillary tangles (NFTs) formed by the microtubule-associated protein tau. Tau filaments adopt multiple morphologies, among which paired helical filaments (PHFs) are the principal constituent of NFTs in the Alzheimer's disease (AD) brain, while straight filaments are a minor variant [2]. In electron micrographs, the PHF appears as a twisted double-helical ribbon of subunits that alternate in width between 10–20 nm and has a half-period of 80 nm [3]. The β-amyloid filaments were known a long time ago to exhibit the characteristic “cross-β” structure, a β-sheet rich structure in which the β-strands are aligned perpendicular to the fibril direction and the interstrand hydrogen bonds are parallel to the fibril axis [4]. However, it is only recently that the “cross-β” characteristics of tau filaments from AD brain and from full-length recombinant protein have been conclusively demonstrated [5]–[7].

Protein tau is primarily expressed in neurons, and is involved in microtubule assembly and stabilization [8],[9]. It is highly soluble and flexible in aqueous solution [10], belonging to the “intrinsically disordered” proteins. Even when it is bound to the surface of microtubules, tau retains most of its disordered character [11]. In adult human brains, there are six isoforms of tau. Depending on the isoform, three or four repeats constitute the core of the microtubule-binding domain. Coincidently, the second and third repeats in the microtubule-binding domain are also the core of PHFs with the cross-β structure, while the rest of the protein forms the fuzzy coat of PHFs [5].

It has been suggested that the motifs VQIINK (PHF6*) in the second repeat and VQIVYK (PHF6) in the third repeat of tau play a key role in the formation of PHF [12],[13]. By transmission electron microscopy (TEM), it was found that AcPHF6 (Ac-PHF6-NH_2_) peptides aggregate into straight filaments [14]. Further, X-ray diffraction patterns and electron micrographs were reported for assemblies of some PHF/tau-related peptides, including AcPHF6 and a longer peptide containing both PHF6* and PHF6 [15]. Assemblies of the latter peptide were found to have a twisted fibrillar structure, whereas the data for AcPHF6 were found to be consistent with a tubular assembly with double walls [15]. An X-ray study of PHF6 microcrystals, on the other hand, found a cross-β spine consisting of β-sheet pairs with a “dry steric zipper” organization at the sheet-sheet interface [16].

Recent findings using transgenic mice models have suggested that soluble aggregated tau rather than NFTs might induce neurodegeneration [17]–[19]. The demonstration of toxicity of soluble aggregates has brought up the possibility of using the oligomeric forms as drug targets. Therefore, it is of great importance to understand the initial nucleation and growth process of tau aggregation. While X-ray diffraction, electron micrography, and microcrystallography have provided information on the structural organization of tau filaments, the initial oligomerization process of full-length protein tau or its peptide fragments remains far from being well understood.

Computational studies have complemented the experiments to provide insights into amyloid formation. Although a wide range of models [20]–[34] has been employed to simulate amyloid aggregation (for a recent review, see [35]), due to the limitations of currently available computer power, most computational studies were limited to small oligomers, short time-scales, or restrained simulations. An alternative approach, to test the stability of preformed structures, has also been explored [36]–[39].

In this work, we study the aggregation of AcPHF6 by an all-atom protein model with a simplified interaction potential using Monte Carlo (MC) simulations. The runs, with up to 36 chains, capture many well known properties of oligomerization. They lead to a multitude of small oligomers rich in β-strand content. We also observe two distinct processes during oligomerization: formation of stable oligomers and emergence of growth capable stable oligomers. Surprisingly, we find that stability of oligomers is not synonymous with their ability to grow. For system sizes permitting the formation of more than one stable oligomer, we find that this type of conformation is more probable than having one large aggregate. Stable oligomers undergo considerable structural reorganization through reptation motion and exchanges of chains with the environment. Growth of stable oligomers is facilitated by a particular kind of ordered structure. New chains do not necessarily attach to a growing oligomer in an ordered manner, so that at every size of the oligomer, there is a slight “barrier” corresponding to a required structural reorganization, before an incremental growth occurs.

## Results

### Oligomerization: Prenucleation Phase

Both experimental [40] and computational [21] works suggest that amyloid formation proceeds via a nucleation process. According to the nucleated conformational conversion model [40], this process shows a two-step behavior: an initial chance association of a sufficient number of monomers to form stable but disordered oligomers, followed by the emergence, through a reorganization process, of ordered oligomers and fibrils. In this article, we begin by studying the first step, i.e., the formation of stable oligomers.

We started with twelve chains of AcPHF6 randomly positioned in the cell, at 308 K. To investigate the concentration dependence, we performed simulations in a number of cubic cells with side lengths of 65 Å, 70 Å, 75 Å, and 80 Å (see Table 1). These concentrations range from 58 mg/ml (73 mM) to 31 mg/ml (40 mM), which are typical values in simulations [30] but higher than the experimental concentrations (0.1–1.0 mg/ml [14]).

**Table 1:** Summary of MC runs.

No. Chains	Edge Length (Å)	No. Runs^a^	Seeded?
12	65	8	No
12	70	50	No
12	75	8	No
12	80	8	No
24	95	72	No
24	95	35	Yes
36	95	72	Yes

a All runs had the same length, 5×10^9^ elementary MC steps.

**Figure 1:**
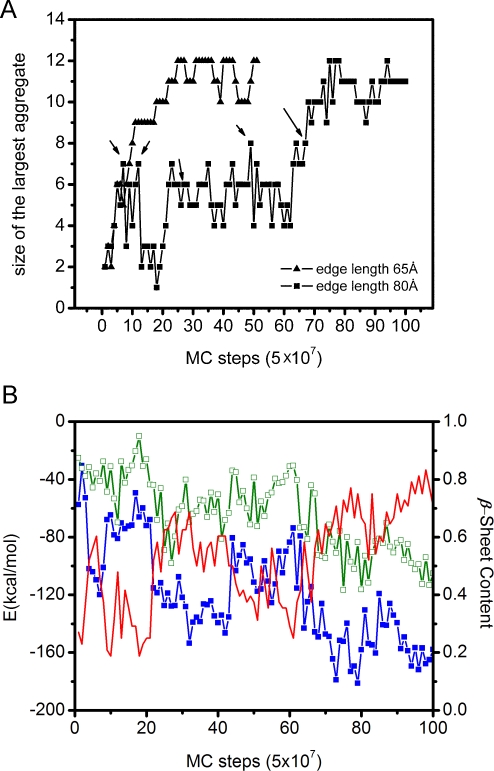
Size of the largest aggregate, energies, and β-sheet content as a function of MC time. (a) The size of the largest aggregate as a function of MC time in two 12-chain simulations for the side lengths 65 Å (triangles) and 80 Å (squares), respectively. A large aggregate forms around step 67 in the run for side length 80 Å and around step 8 in the run for side length 65 Å. Arrows indicate conformations shown in Figure 2. (b) Hydrophobicity energy (in blue), hydrogen bond energy (in green), and β-sheet content (in red) against MC time in the run at low concentration (side length 80 Å). In calculating the β-sheet content, all amino acids of all chains were considered except those at a chain end. The β-sheet content was defined as the fraction of these inner amino acids with their Ramachandran angles in the region 150°<φ<−90°, 90°<ψ<150°.

To identify aggregates in the simulations, we have used a criterion based on contacts between residues belonging to different chains. Two residues were defined to be in contact if the distance between any pair of heavy atoms of these two residues was less than 4.5 Å. Two chains were considered to have a link if they had at least four inter-chain contacts. A set of chains was considered to form a single aggregate, if the graph with those chains as nodes and inter-chain links as edges, was connected. In Figure 1a, we show how the size (number of chains) of the biggest aggregate evolved with MC time in representative runs at the highest (side length 65 Å) and lowest (side length 85 Å) concentrations, respectively (Figure S1 shows the same for six runs with side length 70 Å). In the run at high concentration, aggregation is fast. In contrast, the run at low concentration exhibits a long apparent waiting phase before a large aggregate appears for the first time. In this phase, many meta-stable aggregates with 2–8 chains form and dissolve, without growing into mature stable aggregates. At step 67 (equivalent to 67×5×10^7^ MC steps), a stable aggregate forms for the first time (see below for its conformation), which does not dissolve into smaller aggregates, and the system enters a new, aggregated phase. This behavior is suggestive of a nucleation process, with the nucleation event occurring at step 67. It is worth stressing that the event observed here is nucleation of oligomer formation which is not the same as nucleation of fibril formation. The formation of a critical nucleus for fibrillization generally involves a reorganization process, which might be the rate-limiting step.

Figure 1b shows the evolution of the hydrophobicity energy and the hydrogen bond energy along this (low concentration) trajectory. Both these energies (anti-) correlate with the size of the largest aggregate. The hydrophobic interaction seems to be the most important driving force in the aggregation process, whereas hydrogen bonding also plays a significant role in defining the geometry of the aggregated structures. The aggregation process optimizes both these interactions. Also shown in Figure 1b is the β-strand content, which is strongly correlated with the hydrogen bond energy.

**Figure 2:**
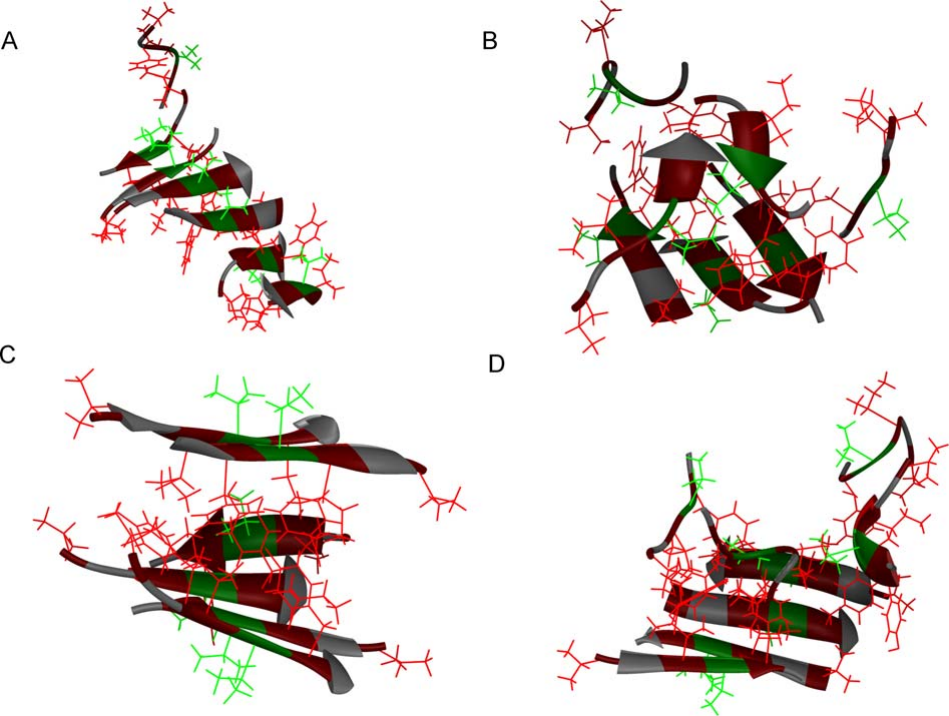
Snapshots of four meta-stable aggregates seen in the pre-nucleation phase of the 12-chain run at low concentration (side length 80 Å). The snapshots were taken at steps 8, 12, 26, and 48, respectively, as indicated by arrows in Figure 1a. V^1^, I^3^ and Y^5^ side chains are colored red, V^4^ side chains are green, and Q^2^ and K^6^ sidechains are, for clarity, omitted. (a) A single β-sheet. (b) A relatively irregular aggregate with two small β-sheets. (c) A small β-sandwich composed of one four-stranded and one two-stranded sheet. (d) A small β-sheet in contact with four random coils.

Figure 2 depicts four examples of meta-stable states from the pre-nucleation phase in the run at low concentration in Figure 1. The first example (Figure 2a) is a six-stranded, mixed parallel/antiparallel β-sheet with a clear twist, in contact with a random coil. Other β-sheets containing 2–6 strands were also observed. The second example (Figure 2b) is a relatively irregular aggregate composed of two small β-sheets with two and three strands, respectively, which are packed against each other. Completely irregular aggregates, without any β-sheet structure, were rare. The third example (Figure 2c) is a small β-sandwich consisting of one two-stranded and one four-stranded β-sheet. Finally, the fourth example (Figure 2d) is a four-stranded β-sheet with four random coils attached to it. All these four aggregates dissolved later and the system remained in the pre-nucleation stage.

According to classical nucleation theory [41], the critical nucleus is in “unstable equilibrium”. The aggregates containing fewer chains than the critical nucleus dissolve spontaneously, while those larger than the critical nucleus grow spontaneously. The system must overcome a free energy barrier and form a critical nucleus before stable aggregates form. This free energy barrier is low when the concentration is high. Indeed, in our simulations, the length of the pre-nucleation phase showed a strong concentration dependence. This is illustrated by Figure 1a, in which nucleation takes place after about 8 steps for side length 65 Å and after about 67 steps for side length 80 Å.

### Oligomerization: Nucleation and Aggregated Phase

**Figure 3:**
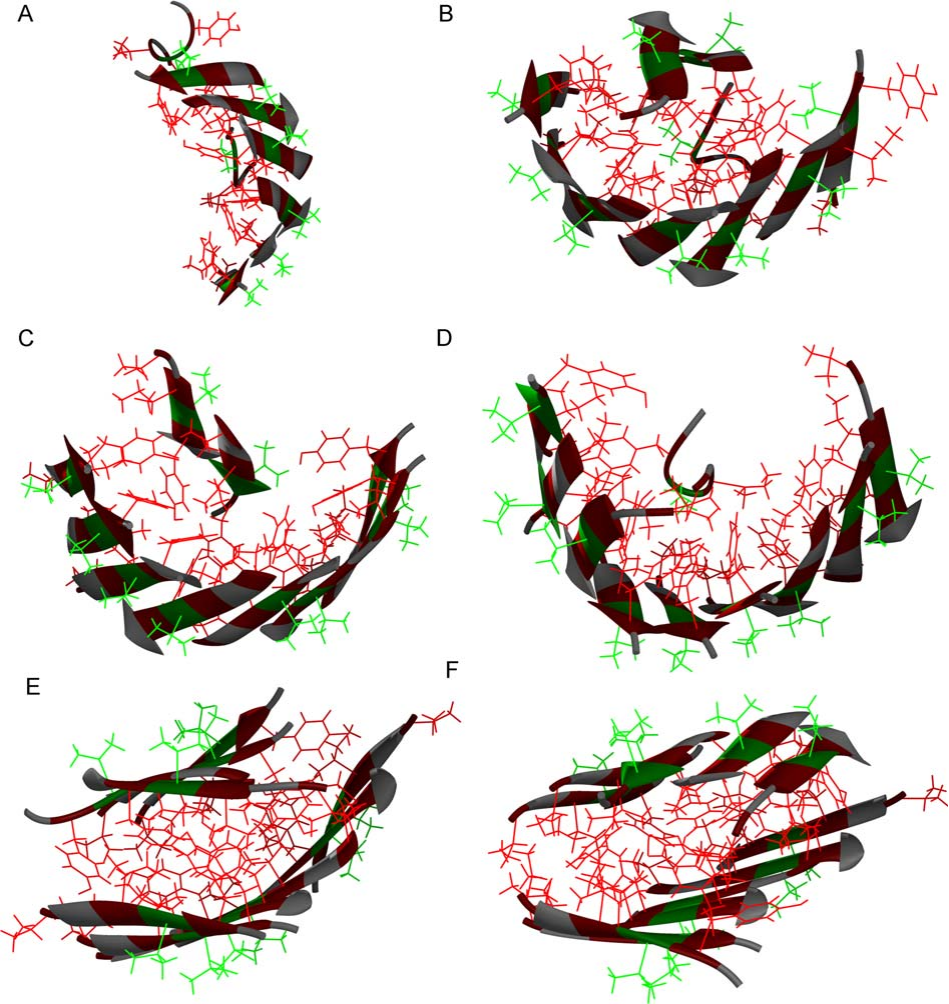
Six snapshots from the aggregated phase of the 12-chain run at low concentration (side length 80 Å). The snapshots (a)–(f) were taken after 67, 68, 71, 86, 91 and 96 steps, respectively. Side-chain colors are as in Figure 2. (a) A six-stranded β-sheet in contact with two random coils. (b) Two β-sheets with seven and two chains, respectively, and a random coil. (c) One eight- and one two-stranded β-sheet. (d) A nine-stranded β-sheet and a 3_10_-helix. (e) One six- and one four-stranded β-sheet. (f) The same sheets as in (e) but with a different relative orientation.

We now illustrate the behavior of the 12-chain system around and after the nucleation step, using the same run as in Figure 2 (side length 80 Å). Figure 3 shows six snapshots from the later part of this run. Once the spontaneous fluctuations result in the formation of a critical nucleus, as at step 67 in this run, the system has reached a point where a stable aggregate may form. The aggregate no longer disperses into smaller pieces or completely dissolves.

At the nucleation event at step 67 (Figure 3a), the aggregate consists of two random coils attached to a twisted six-stranded β-sheet. One step later (Figure 3b), a new chain has joined this aggregate by forming a two-stranded β-sheet with one of the two random coils, and the β-sheet has grown to become seven-stranded. Subsequently, the aggregate undergoes reorganization. At step 71 (Figure 3c), there is one large β-sheet composed of eight chains in contact with a small two-stranded β-sheet. The large sheet is concave, which maximizes contacts with the small sheet. The side chains of V^1^, I^3^, and Y^5^ are buried at the sheet-sheet interface. From this point on, there is a dynamic equilibrium between the aggregate and individual monomers in “solution”. In other words, an individual chain associates with the aggregate or dissociates from the aggregate from time to time, but the total number of peptides within the aggregate does not change significantly with time. Most of the time, the aggregate contains 10–11 chains (Figure 1), but not necessarily the same 10–11 chains at different moments. Individual chains attach and detach at the edges of the β-sheets. Occasionally, the size of the aggregate decreases to nine or increases to twelve chains (Figure 1).

During the dynamic equilibrium, conformational reorganization occurs within the aggregate. Typically, the aggregate consists of two twisted β-sheets wrapped around each other. But the number of chains in each sheet is not constant. We even observed, at step 86 (Figure 3d), a single chain in 3_10_-helix conformation in contact with the concave face of a nine-stranded β-sheet. The most common type of aggregate seen in this run is composed of one five-stranded and one six-stranded β-sheet, as at step 91 (Figure 3e). In sandwich structures like this, large changes in the relative orientation of the two β-sheets were observed. For example, in this run, the angle between the two β-sheets changes from 10° to 60° between step 91 (Figure 3e) and step 96 (Figure 3f). Smaller β-sheets adjust their relative orientation more easily than larger β-sheets. The local bending and the alignment pattern also vary with time. We observed that the alignment of the edge strands of a β-sheet can change without their detachment from the β-sheet. However, the alignment pattern in the central part of a β-sheet were not seen to change, which is understandable as there are constraints from neighboring strands.

Structures resembling those in Figure 3 have been observed in previous simulations of smaller systems, including 6-chain simulations with explicit water for other peptides of the same length as PHF6 [28]. The curved β-sheets seen in Figure 3b–d are reminiscent of the open β-barrels reported by Derreumaux and coworkers [42],[43].

The structures shown in Figures 2 and 3, all from a single run, illustrate some general features seen in all our simulations. For example, a vast majority of our observed aggregates are β-sheet rich, and both curved sheets and sandwich-like structures are frequently occurring motifs. However, the details of the structures in Figures 2 and 3, like the exact alignment of the β-strands, are not statistically representative.

To statistically characterize aggregated structures, we carried out an additional set of fifty 12-chain runs, starting from different random initial configurations. For computational convenience, the side length was here set to 70 Å instead of 80 Å. In these runs, the same two phases were seen as in the 80 Å run described above, but the aggregation process was faster. In seven of the fifty runs, the aggregate converted to a stable β-barrel containing 8–12 chains. This type of conformation was not further investigated, because the main focus of the present study is aggregate growth, and a β-barrel is unlikely to grow into a larger aggregate.

**Figure 4:**
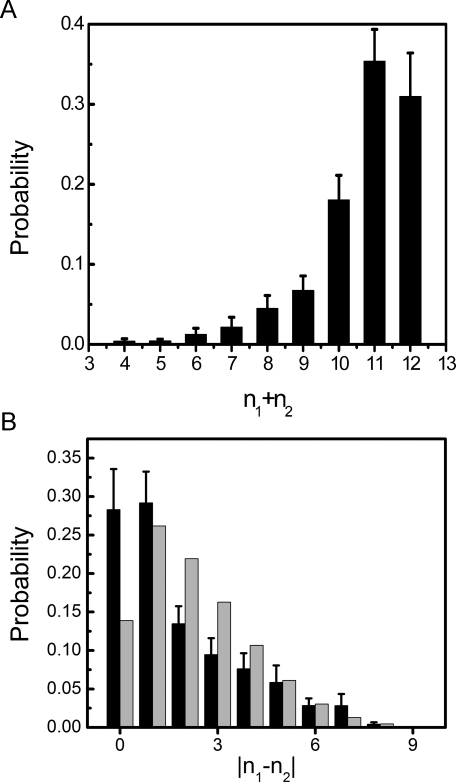
Size analysis of two-sheet aggregates in our fifty 12-chain runs for a side length of 70 Å. Only the second half of the trajectories was used for the analysis. n_1_ and n_2_ denote the numbers of chains in the two sheets of a given aggregate. (a) Observed distribution of the total number of chains, n_1_+n_2_. (b) Observed distribution of the difference in number of chains, |n_1_−n_2_| (in black). Also shown, for comparison, is a purely combinatorial estimate (in grey). It represents the probability for a certain difference in the sizes of the first two groups when a set of 12 objects are divided randomly into 3 groups.

For sandwich structures, we made a size analysis based on these fifty runs. Here we counted the total number of chains in the aggregate and the difference in number of chains between the two β-sheets after a stable two-sheet aggregate had formed. Figure 4a shows the observed distribution of aggregate size. The peak is at 11, whereas aggregates with ≤7 chains are quite rare. Note that the distribution depends on the concentration. Figure 4b illustrates the difference in size between the two β-sheets in two-sheet aggregates. Small size differences of 0 or 1 are most common. We note that whenever any collection of objects is randomly divided into two groups, there are more ways of constructing the groups with nearly equal size than of constructing them with a large size difference. Therefore large size differences would be expected to be suppressed entirely due to combinatorial considerations, irrespective of the specific properties of the system. In Figure 4b, the largest size differences involve one very small aggregate, and the observed probabilities are nearly consistent with the random estimate. But the probability of the smallest size differences is enhanced at the expense of the medium size differences of 2 and 3 in Figure 4b, suggesting that size symmetry of sheets in an oligomer is further favored due to interactions.

### The Interplay between Hydrogen Bonding and Hydrophobic Interactions

**Figure 5:**
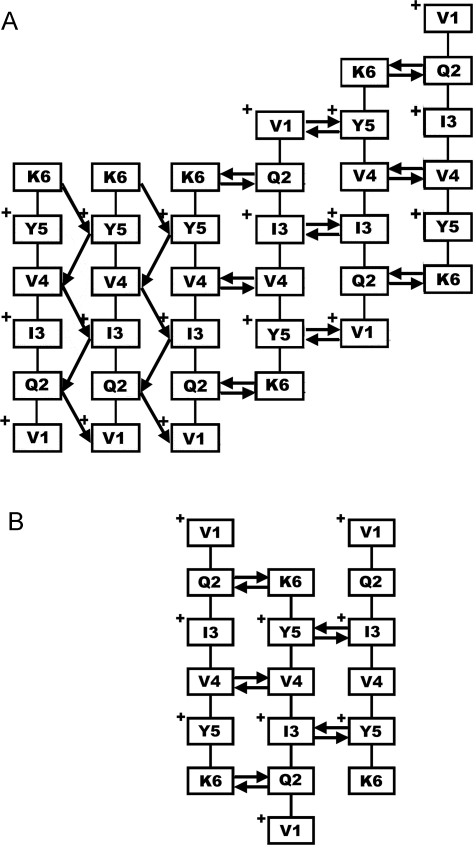
Illustration of parallel and anti-parallel arrangements. (a) Hydrogen bond pattern and strand alignment for the largest of the two β-sheets in Figure 3e. A “+” sign indicates that the side-chain is pointing outwards. (b) Illustration of an antiparallel off-register arrangement leading to a zig-zag pattern. Note that, despite the apparent symmetry about strand two, the number of hydrogen bonds between strands two and three is not the same as that between strands one and two. The arrows indicate hydrogen bonds, and point from the donor to the acceptor.

Inspection of the aggregates in Figure 3 shows that the β-strands tend to be arranged so that there are many hydrophobic intra-sheet contacts between them, involving the V^1^, I^3^ and Y^5^ side chains. A vast majority of the larger aggregates seen in our simulations share this property. In order to maximize the hydrophobic interaction between two adjacent strands in a sheet, the V^1^, I^3^, and Y^5^ side chains of both strands must point to the same side of the sheet (and Q^2^, V^4^, and K^6^ to the other). This is achieved if the strands are parallel and either in-register or off-register by two residues, or if they are antiparallel and off-register by one or three residues. Obviously, this property depends on sequence. For example, if the peptide has an alternating hydrophobic/hydrophilic pattern but an odd number of residues, both parallel and antiparallel in-register arrangements will maximize the hydrophobic interactions. Figure 5a illustrates the strand organization and the orientation of the side chains for the largest of the two β-sheets in Figure 3e. The strand organization is such that all V^1^, I^3^, and Y^5^ side chains point to the same direction.

In our simulated aggregates, there were two dominating β-strand arrangements, illustrated by Figure 5a, namely parallel in-register and antiparallel out-of-register by one residue. These arrangements lead to different hydrogen bond patterns. With a parallel in-register arrangement, each pair of adjacent strands is connected by five hydrogen bonds (see Figure 5a). If two strands are antiparallel and off-register by one residue, there will be either four or six hydrogen bonds connecting them. The arrangement can be repeated so that there are six hydrogen bonds between all pairs of adjacent strands (see Figure 5a), with successive strands shifting in the same direction. Another possibility is that the third chain is in the same relation to the second as the second is to the first, which leads to a zig-zag pattern with successive strands shifting in opposite direction, as in Figure 5b. The drawback of this organization is that there are only four hydrogen bonds between the second and the third strand. We observed this pattern in our simulations, but with a relatively low frequency. The organization to the right in Figure 5a, which maximizes the number of hydrogen bonds, was more common. Unlike both these organizations, the parallel organization, to the left in Figure 5a, is in-register, which may be advantageous in large sheets. In fact, we found that the fraction of parallel over antiparallel β-sheet structure increased with aggregate size, as will be discussed below.

### Oligomer Growth

Having observed the formation of stable oligomers in the 12-chain runs, we increased the system size to 24 chains to study oligomer growth. For this system size, we performed a set of 72 unseeded and 35 seeded runs (see Table 1), at the same temperature as before (308 K). The side length was 95 Å, corresponding to a concentration of 36.7 mg/ml.

**Figure 6:**
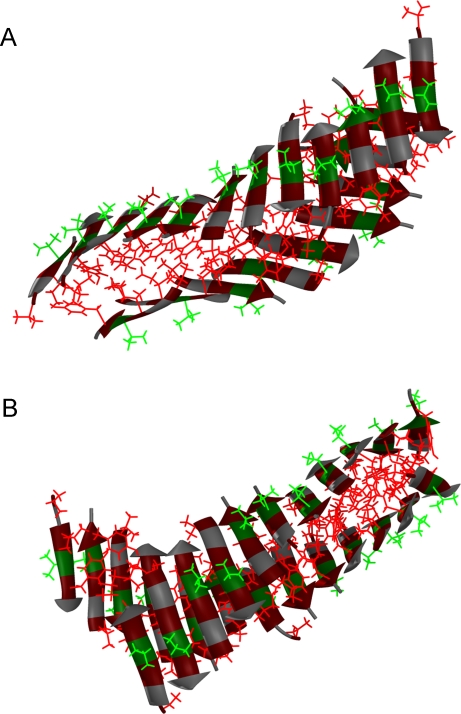
Snapshots of two large aggregates from (a) an unseeded and (b) a seeded 24-chain run. Side-chain colors are as in Figure 2.

In our unseeded 24-chain runs, the same two phases were observed as in the 12-chain runs: an initial waiting phase with only small aggregates, followed by a phase with large aggregates in dynamic equilibrium with small aggregates and free monomers. The waiting phase was often short, due to the relatively high concentration. In the aggregated phase, there was in some runs one dominating aggregate, typically with 16–20 chains. These big aggregates were invariably composed of two stacked β-sheets, as in Figure 6a. In a majority of the runs, there were two, rather than one, major aggregates, each with typically 8–10 chains. These aggregates were similar to those observed in our 12-chain runs. Some of them were of the barrel type. In total, we saw 15 stable β-barrels in our 72 unseeded 24-chain runs. As in the 12-chain case, these aggregates, which are unlikely to grow into larger aggregates, will not be further analyzed in this work.

**Figure 7:**
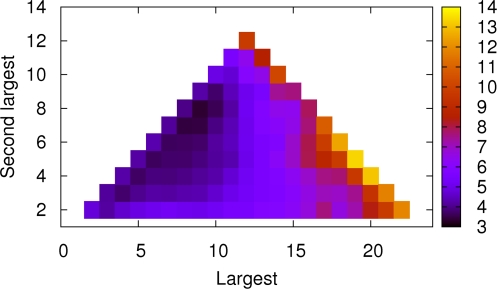
Statistical analysis of aggregate sizes in our 72 unseeded 24-chain runs. The quantity shown is −log H(x,y), where x and y denote the sizes of the largest and next-largest aggregates, respectively, in a given conformation; H(x,y) is the histogram of x and y. Conformations were recorded at regular time intervals in the second half of the runs.

To get a quantitative picture of the aggregation behavior of the 24-chain system, we determined the sizes of the largest and next-largest aggregates for each conformation in our unseeded runs, which we denote by x and y, respectively. Figure 7 shows minus the logarithm of the histogram of x and y. The most populated region corresponds to two aggregates of similar size, 8–10 chains. In addition, there is a weak local minimum at large x and low y, corresponding to configurations with one dominating aggregate. However, the minimum corresponding to two distinct similar size aggregates is much deeper. If the growth of an arbitrary stable oligomer had been easy, upon the formation of the first such oligomer, the remaining free monomers would have rapidly associated with that, leading to one big aggregate. But in Figure 7 we see that the probability of two similar-size aggregates is significantly larger. This indicates that further growth is not fast compared to the formation of a new nucleus. Instead, at a size of ∼10 chains, it seems that the aggregation process reaches a stage at which conformational reorganization is required before further growth.

Our 35 seeded 24-chain simulations further elucidate this point. These runs were started from initial conformations prepared by taking aggregates from the 12-chain runs and adding random coils. Despite the presence of a template, a large aggregate (with >15 chains) appeared in only 15 of these runs. In 17 of the remaining runs, the free monomers instead assembled into a second aggregate, thus leading to a state with two distinct aggregates of similar size. In the remaining 3 runs, the newly added chains stayed in the pre-nucleation phase. Even when a stable seed is present, an independent new aggregate thus forms in about half of our runs, which suggests that the time scale for the conformational reorganization required for further growth is comparable to that of the formation of a new stable aggregate of about 10 chains.

Figure 6b gives an example of a large aggregate from a seeded simulation. After the first large aggregate had appeared in this run, the system spent the rest of the run in a phase of dynamic equilibrium, with only small fluctuations in aggregate size. Figure 6b shows a randomly chosen snapshot from this phase. This aggregate shares several common features with that from the unseeded simulation shown in Figure 6a. Both aggregates are composed of two large β-sheets that are twisted and wrap around each other. The overall twist is 8.8° and 6.3° for the two sheets in Figure 6a, and 12.0° and 13.9° for those in Figure 6b (see Methods). In both structures, parallel strand pairs are either in-register or out-of-register by two residues, while antiparallel pairs are off-register by one residue, as discussed in connection with Figure 5. In addition to being twisted, the strands are also bent, which helps to make better side-chain contacts. Twisting, bending, strand alignment and side-chain packing are all important factors influencing the final conformation.

**Figure 8:**
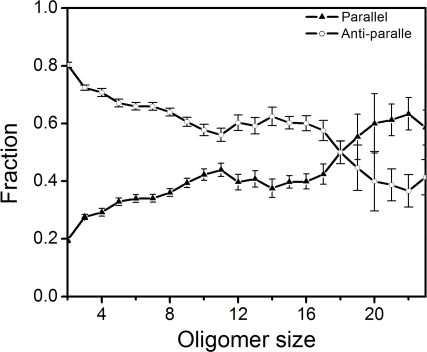
The fractions of parallel and antiparallel β-sheet structure against aggregate size, as obtained from our unseeded 24-chain runs. The set of conformations used for the analysis is the same as in Figure 7.

Based on our 72 unseeded 24-chain runs, we performed a statistical analysis of some important properties of the aggregated structures. Of particular interest is the β-sheet organization, which in most of our simulated aggregates is mixed parallel/antiparallel (see Figures 2, 3, and 6) but is known to be parallel in AcPHF6 fibrils [14]. In our 72 runs, we counted parallel and antiparallel pairs of adjacent strands for all aggregates of a given size. Figure 8 shows the fractions of parallel and antiparallel β-sheet structure, as obtained this way, against aggregate size. For small sizes, there is a clear statistical preference for the antiparallel organization. However, the fraction of parallel structure increases steadily with aggregate size. In aggregates with more than 18 chains, the parallel organization is more common than the antiparallel one.

**Figure 9:**
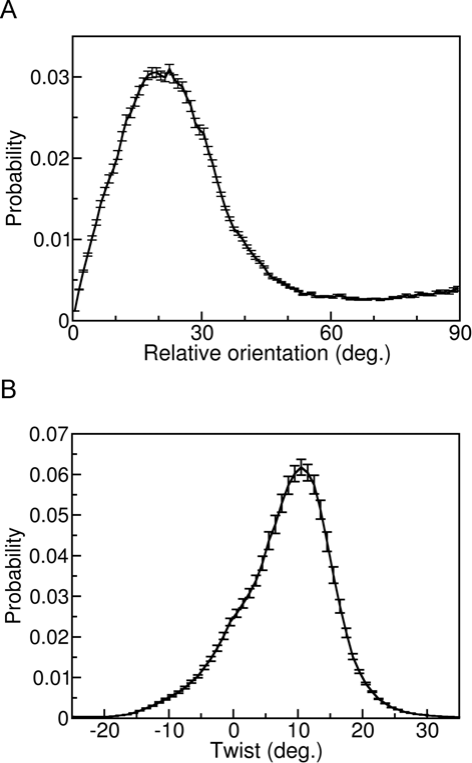
Structural properties of β-sandwiches in our unseeded 24-chain runs. The set of conformations analyzed is the same as in Figure 7. (a) Distribution of the relative orientation of the two β-sheets in two-sheet aggregates with 5–7 strands in each sheet. (b) Distribution of sheet twist for β-sheets in two-sheet aggregates with at least five strands in each sheet. A positive sign of the twist angle indicates a left-handed twist about the in-sheet axis perpendicular to the peptide chain.

Using the same runs, we also analyzed the relative orientation of the two β-sheets in sandwich structures. For all two-sheet structures with 5–7 chains in each sheet, the angle between the two sheets was determined (see Methods). Figure 9a shows the calculated distribution of this angle. The distribution exhibits a broad peak in the region 5°–35°. Relative orientations in the range 45°–90° occur but are rare.

Finally, we also calculated the overall twist for all β-sheets in two-sheet aggregates with at least five strands in each (see Methods). The two edge strands of a sheet were not included in the analysis, because we found that those strands often were more twisted than the rest of the sheet. Figure 9b shows the observed distribution of the average twist angle. Its maximum is near 11°. The shape of the distribution is asymmetric, with a shoulder near 0°.

It is worth pointing out that the runs presented in this article are of limited length. In any given run, it is most likely that some important free-energy minima were not sampled, due to high free-energy barriers. Our findings are, however, based on a set of many independent simulations. We feel confident that major trends seen, like the increase in the fraction of parallel β-sheet structure with aggregate size, are statistically robust.

We also did seventy-two 36-chain simulations (see Table 1), seeded with minimum energy conformations from our 72 unseeded 24-chain runs, to which 12 random coils were added. In many of these runs, the formation of a new independent aggregate with ∼10 chains was observed, whereas the one or two major aggregates present initially grew by only one or a few chains. In a few runs, significant growth was observed for one of the pre-existing seeds. The behavior of the 36-chain system thus supports the picture emerging from the 24-chain runs. New independent aggregates large enough to be long-lived form relatively easily in our simulations, but that an aggregate reaches this size does not mean that further growth is fast. Most aggregates seem to require conformational reorganization before they can grow, which prevents rapid growth. A detailed analysis of our 36-chain runs is beyond the scope of the present work and will be presented in a forthcoming publication.

## Discussion

### The Formation of Stable Oligomers

As of now, the nucleation event in the oligomerization process is difficult or impossible to examine using experimental approaches. Light and neutron scattering techniques are capable of revealing the shape of micelles of Aβ, but these aggregates are larger than the critical nucleus [44]. Computer simulations offer unique opportunities to observe and analyze early aggregation events at the molecular level.

In our 12-chain simulations of AcPHF6 peptides from random initial conformations, two distinct phases can be identified: an early phase with only smaller aggregates, followed by a phase characterized by the presence of a large aggregate. This behavior suggests that the formation of stable oligomers occurs through a nucleation process. In a simple nucleation process, an embryo of the new phase will grow spontaneously once it has reached a certain critical size. By contrast, when proteins/peptides aggregate, the size of the embryo is not the only relevant parameter; the ability of the critical embryo to grow depends also upon its conformation. Our run at low concentration in Figure 1 illustrates this. Here the nucleation event occurred after 67 steps. Some aggregates of comparable size appeared before step 67, but these aggregates dissolved into smaller aggregates and/or monomers. The pre-nucleation behavior of our system resembles what has been seen in several previous studies of small systems with 2–9 chains. These studies found many different meta-stable aggregates with various forms of β-sheet structure, and suggest that the barrier for converting from one of these aggregates to another is low [26], [37], [45]–[48].

The aggregate observed at the nucleation stage in the run discussed above (step 67) is composed of a twisted six-stranded β-sheet and two attached random coils (Figure 3a). A striking feature of this β-sheet is that the V^1^, I^3^, and Y^5^ side chains point in the same direction in all strands. The aggregates from the pre-nucleation phase (see Figure 2) do not have this property. In the proposed dry steric zipper model for PHF6 fibrils, based on X-ray microcrystallography, the β-sheet pair adopts a face-to-face stacking arrangement in which the side chains of V^1^, I^3^, and Y^5^ nestle between sheets [16]. The V^1^, I^3^, and Y^5^ side chains pointing to the same side of a β-sheet might very well be a prerequisite for its participation in a stable double-layered structure. Even a single strand with the V^1^, I^3^, and Y^5^ side chains pointing to the opposite side of the β-sheet could mean that the entropy loss upon formation of the aggregate cannot be compensated by an enthalpy reduction from intermolecular interactions.

The second component of this aggregate (Figure 3a) is the two random coils that are in contact with the β-sheet. One of the random coils is attached to the V^1^–I^3^–Y^5^ side of the sheet, solely by side-chain interactions, while the other is attached to an edge of the sheet. This kind of arrangement has been observed in previous simulations for Aβ_16–22_ [45], Aβ_11–25_ [49], and the GNNQQNY segment of yeast prion protein Sup35 [31],[50]. The attachment of random coils to a β-sheet might help to stabilize the sheet through side-chain interactions.

In the aggregated phase, after the nucleation event, we observed different kinds of β-sheet rich aggregates. A common type of conformation was the β-sandwich. In these aggregates, the two sheets tended to be of similar size (Figure 4), and V^1^, I^3^ and Y^5^ side chains were typically found at the sheet-sheet interface (see Figure 3c, 3e, and 3f), as in the dry steric zipper arrangement [16].

An interesting question is how ordered early oligomers are. Nguyen and Hall employed a coarse-grained protein representation and discontinuous molecular dynamics to simulate amyloid aggregation for a system of 96 polyalanine peptides (Ac-KA_14_K-NH_2_). As a first step, preceding fibrillization, the chains were found to form irregular aggregates, which then converted into small β-sheets [21]. In our simulations, very few completely disordered aggregates were found. In part, this may be due to the short length of our peptide, which leads to a high propensity for it to be in an extended state. Another factor influencing the aggregation into either ordered or amorphous species is the hydrophobicity of the sequence. Indeed, a recent study by Cheon et al. [30] found amorphous aggregates for the more hydrophobic Aβ_16–22_ peptide but only ordered aggregates for the less hydrophobic Aβ_25–35_ peptide. Our results suggest that AcPHF6 behaves like Aβ_25–35_ rather than like Aβ_16–22_ in this respect.

### Twist and Relative Orientation of β-Sheets

The large aggregates seen in our study also differ from the aggregates seen in the polyalanine simulations of Nguyen and Hall [21]. One difference is that the β-sheets lacked twist in the polyalanine study. Another, possibly related, difference is that Nguyen and Hall saw multiple-sheet stacking, whereas our aggregates were sandwich structures with only two stacked β-sheets. The relation between sheet stacking pattern and sheet twist was studied in recent simulations [37].

β-sheets in the Protein Data Bank (PDB) are generally twisted. A recent study analyzed β-sheet twist in PDB structures in terms of adjacent pairs of residues on neighboring β-strands [51]. The average twist angle was found to be 17°±7° for parallel β-sheets, 15°±10° for non-hydrogen bonded residue pairs in antiparallel β-sheets, and 8°±8° for hydrogen bonded residue pairs in antiparallel β-sheets [51]. The distribution of sheet twist angle presented in Figure 9b is in line with the above statistics. Sheet twisting has also been observed in explicit-water molecular dynamics studies of preformed β-sheets [31],[36],[37],[39]. For a pair of ten-stranded β-sheets of the peptide GNNQQNY, the average twist within each sheet was found to be about 11° after a 20 ns simulation [36] using GROMACS [52], which is in excellent agreement with our results shown in Figure 9b. That the sheet twist found in this study as well as in ours is comparable to that of native proteins is not surprising, because the aggregates were relatively small.

For native proteins, there is a tendency that β-sheets with few strands are associated with larger twist angles than those containing a large number of strands [53]. From this observation, one might expect the sheet twist to be smaller in amyloid fibrils than in native proteins. This expectation is supported by data from cryo-electron microscopy experiments on insulin fibrils (twist angle 1.5°–2.5°) [54] and TEM experiments on fibrils of rationally designed peptides (twist angle 1°–3°) [55]. Solid-state NMR data on TTR_105–115_ [56] and Aβ_1–40_ [57] fibrils were found [58] to lead to slightly larger twist angles (26°±24° for TTR_105–115_, 14°±37° and 17°±38° for two Aβ_1–40_ data sets), but with large statistical uncertainties.

One parameter in describing a two-sheet aggregate is the relative orientation of the sheets. For small aggregates, one might expect large variations in this parameter. In our simulations, we measured an angle describing the relative orientation of the sheets. The distribution of this angle was indeed found to be broad (Figure 9a). Further, we saw large rotations of sheets relative to each other during the course of our simulations. For example, a ∼50° rotation occurred between steps 91 and 96 in one of the runs (Figure 3e and 3f). This rearrangement of the aggregate is concurrent with a reduction of both the hydrophobic interaction energy and the hydrogen bond energy (Figure 1b). Large relative rotations of β-sheets have also been seen in explicit-water molecular dynamics simulations for Aβ_16–22_ [37], using the AMBER/parm99 force field [59]. After 20 ns, a pair of preformed β-sheets had rotated by ∼90° relative to each other, leading to a better packing and stronger hydrophobic interactions. These observations of large β-sheet rotations in simulations based on completely different models indicate that this kind of movement is common in small aggregates.

### Aggregate Growth

To study further growth after the formation of a stable oligomer, we performed a large set of unseeded 24-chain runs. In some of these runs, a large aggregate with ∼20 chains formed, However, in most runs, the chains formed two aggregates of similar size rather than one big one (Figure 7). Even when a stable seed was present, an independent new aggregate with ∼10 chains appeared in a about half of the runs. These results indicate that while oligomers with ∼10 chains easily form in this system, many of them are not growth-competent; further growth seems to require time-consuming conformational rearrangement.

Since virtually all oligomers seen in our simulations had a large fraction of β-sheet structure, we further conclude that for a given oligomer to be able to grow, it is not sufficient that it has a high β-sheet content; the organization of the β-strands is also important. Interestingly, we found a correlation between aggregate size and the ratio of parallel to antiparallel β-sheet structure (Figure 8). Antiparallel structure was most common for small aggregates, but the fraction of parallel structure increased steadily with aggregate size (Figure 8). The β-strand organization in AcPHF6 fibrils is known from experiments to be parallel. FTIR (Fourier Transform Infrared) spectra showed amide I bands with maxima at 1619 cm^−1^ and 1647 cm^−1^, characteristic of a parallel β-strand arrangement, while no high frequency component corresponding to an antiparallel arrangement was found [14].

Could the aggregates we observe be kinetic traps en route to fibrils, or are they more likely to be off-pathway states? This depends on the time scale for the reorganization process and cannot be answered based on our current data. Most β-sheets we observe contain some strands far from the edges whose orientation must change for the sheet to become parallel. This could, in principle, occur through breaking and joining of β-sheets [60], but whether that is viable mechanism for the system studied here is unclear. Another mechanism is repeated attachment/detachment of edge strands. The time scale for changing the orientation of a central strand by this mechanism is, however, unknown.

The conformational reorganization of soluble β-sheet aggregates, toward more ordered structure, has been investigated experimentally by Decatur and coworkers using isotope-edited FTIR spectroscopy for Aβ_16–22_ and H1, a 14-residue fragment of the prion protein [61]–[63]. Two competing mechanisms of reorganization were proposed: the detachment-reattachment of strand(s) from/onto existing β-sheets, which was found to dominate at low concentration, and the sliding or reptation of individual strands without detachment from the aggregate, which was found prevalent at high concentration. The reptation motion has been observed in simulations for Aβ_16–22_ [22],[64], TTR_105–115_ [32] and GNNQQNY [65]. Both the above mechanisms of β-sheet reorganization were seen in our simulations, along with large-scale motion of whole β-sheets relative to each other.

The time required for the conversion of early formed aggregates into growth-competent ones depends on the character of the early aggregates, and therefore on sequence. The process need not be faster if the early aggregates are β-sheet rich, because the system then has to escape from deep unwanted minima. Our results suggest that β-sheet rich aggregates form fast for AcPHF6, but the reorganization needed for further growth may be slow. This finding is consistent with the Aβ_16–22_ and H1 results of Decatur and coworkers [61]–[63], although the precise character of the reorganization process may have been different in their systems. For AcPHF6, we find that changes in the parallel/antiparallel organization are an important part of the reorganization process.

While our simulated aggregates show features reminiscent of the proposed dry steric zipper model [16] for AcPHF6 fibrils, we found no indication that a nanotubular structure [15] would emerge with increasing aggregate size. It must be kept in mind, however, that there is a huge gap in size between our simulated aggregates and full fibrils.

### Conclusion

We have carried out extensive seeded and unseeded Monte Carlo simulations of the aggregation of the peptide AcPHF6, derived from the tau protein, using an all-atom protein model with a simplified interaction potential. Our results suggest that the formation of stable AcPHF6 oligomers occurs through a nucleation process. In the pre-nucleation phase, a variety of meta-stable aggregates formed and dissolved. At the nucleation stage, the aggregates had already acquired a large fraction of β-sheet structure; no completely disordered aggregates of significant size were seen in our simulations. The oligomers formed in this nucleation step are thus β-sheet rich, but they are not necessarily growth-competent. Our results indicate that further growth requires conformational reorganization. The reorganization process appears to be slow, and might be the main bottleneck to fibril formation for this peptide.

In some runs, large aggregates appeared, with ∼20 chains or more. All these aggregates were composed of two twisted β-sheets, packed against each other with V^1^, I^3^ and Y^5^ side chains forming the sheet-sheet interface. This kind of conformation bears a striking resemblance to the dry steric zipper structure that has been proposed for PHF6 fibrils [16]. Morover, while most aggregates we saw had a mixed parallel/antiparallel β-strand organization, there was a clear tendency that the fraction of parallel β-sheet structure increased with aggregate size. In the fibrils, the β-strand organization is known to be parallel [14]. From these observations, it is tempting to speculate that reorganization of the β-sheets into parallel ones is a key step in the formation of PHF6 fibrils.

## Methods

### All-Atom Minimalistic Model

The package PROFASI (PROtein Folding and Aggregation SImulator) [66] was employed in this work. The model is an implicit water all-atom description (including all hydrogen atoms) of the protein chains with only torsional degrees of freedom (without bond stretching and angle bending). In addition to these, each chain has three translational and three rotational degrees of freedom. The interaction energy of the model is
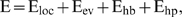
where E_loc_ is a local electrostatic interaction between adjacent peptide units which influences the Ramachandran φϕ distribution, and the others are non-local terms. The excluded volume term E_ev_ represents an r^−12^ repulsion between atom pairs. E_hb_ is an explicit hydrogen-bond term modeling backbone-backbone and charged side chain-backbone hydrogen bonds. E_hp_ describes an effective hydrophobic interaction between pairs of non-polar side chains which depends on the degree of contact of the two side chains. The details of each interaction term and the corresponding parameters can be found elsewhere [67],[68]. Whereas it is a minimalistic model, with the potential deliberately kept simple for the sake of clarity and computational efficiency, the model has successfully captured the folding thermodynamics and kinetics of peptides and small proteins, peptide aggregation, and the mechanical unfolding of a 76-residue protein [20], [45], [67]–[70].

### MC Details

All the simulations were carried out in a cubic cell with periodic boundary conditions at a constant temperature. The temperature was set to 0.46 in reduced units, corresponding to ca 308 K, for optimal computational efficiency. If the temperature is too high, the chains will not aggregate, while if the temperature is too low, the kinetic evolution of the system will be slow. The temperature studied is close to the experimental conditions [14].

The conformational MC updates included chain translations (6.65%), chain rotations (6.65%), single-variable updates of side-chain (51.0%) as well as backbone (26.6%) angles, and Biased Gaussian Steps that favor local deformations of the protein [71] (9.1%). The results did not change when we slightly modified the relative frequencies of the different moves, for example, to (6.1%, 6.1%, 46.7%, 24.4%, 16.7%) or (7.3%, 7.3%, 56.1%, 29.2%, 0.0%). Note that none of these updates move more than one chain at a time. The conformations were saved every 10^3^ MC steps. Since the peptide is very short, a single backbone torsion angle change does not yield drastic changes in the global structure as it would for long peptide chains. For this reason, we believe that for this peptide, the MC dynamics mimic the random motions of the peptides and can be interpreted as a discrete form of Brownian dynamics [72], so that the events along the Markov chain in our MC simulation can be considered as a coarse-grained dynamic process. For conciseness, the unit of simulation time is 50 million MC steps in this article, so 1 step is equal to 5×10^7^ MC steps unless noted otherwise. Note that one cannot compare the reaction rates of systems that have different numbers of degrees of freedom using the number of MC steps directly. When the number of degrees of freedom is doubled, to represent the same time scale, the required MC steps should also be doubled.

We performed both unseeded runs started from random initial conformations, and seeded runs, where the initial conformations contained aggregates from simulations of smaller systems. Our runs are summarized in Table 1. All statistical errors quoted were obtained by the jackknife method [73].

### Structural Analysis

For the analysis of the simulation data, we defined the end-to-end vector of a chain as the one from the first backbone N atom to the last backbone C atom. When the acute angle between two normalized end-to-end unit vectors was <30° and the interstrand main-chain hydrogen bond energy was <−6.1 kcal/mol (corresponding to 2–3 hydrogen bonds), the two chains were considered to form a sheet. A pair of strands was defined as parallel (antiparallel) if the dot product of their normalized end-to-end vectors was between 0 and 1 (−1 and 0). To define the direction of a sheet, we used the average of all end-to-end vectors within the sheet, calculated after reversing antiparallel end-to-end vectors to make all vectors roughly point in the same direction. The relative orientation of two sheets was calculated as the acute angle between the direction vectors of the sheets. To describe the twist of a β-sheet, we defined the twist angle between pairs of adjacent strands in the sheet as the acute angle formed by the backbone direction vectors of two strands. The backbone direction vector was taken to start at the middle point of the peptide bond between Q^2^ and I^3^ and end at the middle point of the peptide bond between V^4^ and Y^5^. The first and last residues were omitted because they were often unstructured. The average twist angle within a sheet indicates the overall twisting of the sheet. A positive sign of the twist angle indicates a left-handed twist about the in-sheet axis perpendicular to the peptide chain. It corresponds to a right-handed twist about the axis running in the same direction as the peptide chain.

## Supporting Information

Figure S1 MC time evolution of the size of the largest aggregate in six 12-chain runs with side length 70 Å.
(0.48 MB TIF)
