# Supplementary figures and images for: Formation and Growth of Oligomers: A Monte Carlo Study of an Amyloid Tau Fragment
Source: PLoS Comput Biol. 2008 Dec 5;4(12):e1000238. doi: 10.1371/journal.pcbi.1000238 (PMC2583953; doi:10.1371/journal.pcbi.1000238)

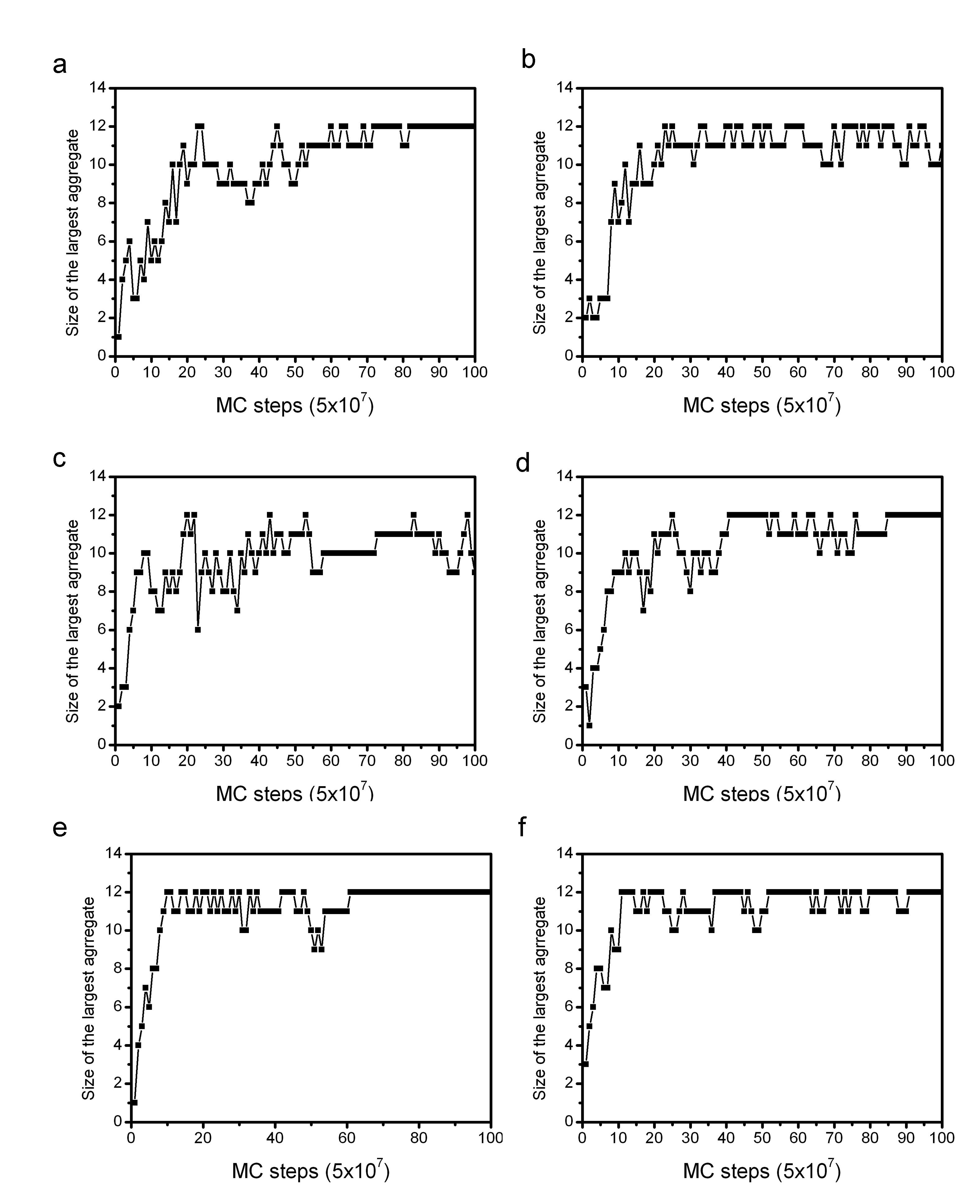

Supplement: Figure S1 — MC time evolution of the size of the largest aggregate in six 12-chain runs with side length 70 Å. (0.48 MB TIF) [file pcbi.1000238.s001.tif]
